# Biotic and abiotic effects of soil organic matter on the phytoavailable phosphorus in soils: a review

**DOI:** 10.1186/s40538-023-00401-y

**Published:** 2023-03-28

**Authors:** Keiji Jindo, Yuki Audette, Fabio Lopez Olivares, Luciano Pasqualoto Canellas, D. Scott Smith, R. Paul Voroney

**Affiliations:** 1 Agrosystems Research, Wageningen University & Research, Wageningen, 6700AA The Netherlands; 2 School of Environmental Sciences, University of Guelph, Guelph, ON N1G 2W1 Canada; 3 Chitose Laboratory Corp., Kanagawa, 213-0012 Japan; 4 Laboratório de Biologia Celular e Tecidual & Núcleo de Desenvolvimento de Insumos Biológicos para Agricultura, Universidade Estadual do Norte Fluminense Darcy Ribeiro, Campos dos Goytacazes, Rio de Janeiro, 28013-602 Brazil; 5 Department of Chemistry and Biochemistry, Wilfrid Laurier University, Waterloo, ON N2L 3C5 Canada

**Keywords:** Phosphorus, Organic matter, Phytoavailable P, Sorption/desorption, Metal complexation/dissolution, Ternary complex, Mineralization, Enzyme, P solubilizing microorganisms

## Abstract

Soil organic matter (SOM) has a critical role in regulating soil phosphorus (P) dynamics and producing phytoavailable P. However, soil P dynamics are often explained mainly by the effects of soil pH, clay contents, and elemental compositions, such as calcium, iron, and aluminum. Therefore, a better understanding of the mechanisms of how SOM influences phytoavailable P in soils is required for establishing effective agricultural management for soil health and enhancement of soil fertility, especially P-use efficiency. In this review, the following abiotic and biotic mechanisms are discussed; (1) competitive sorption between SOM with P for positively charged adsorption sites of clays and metal oxides (abiotic reaction), (2) competitive complexations between SOM with P for cations (abiotic reaction), (3) competitive complexations between incorporation of P by binary complexations of SOM and bridging cations with the formation of stable P minerals (abiotic reaction), (4) enhanced activities of enzymes, which affects soil P dynamics (biotic reaction), (5) mineralization/immobilization of P during the decay of SOM (biotic reaction), and (6) solubilization of inorganic P mediated by organic acids released by microbes (biotic reaction).

**Graphical Abstract:**

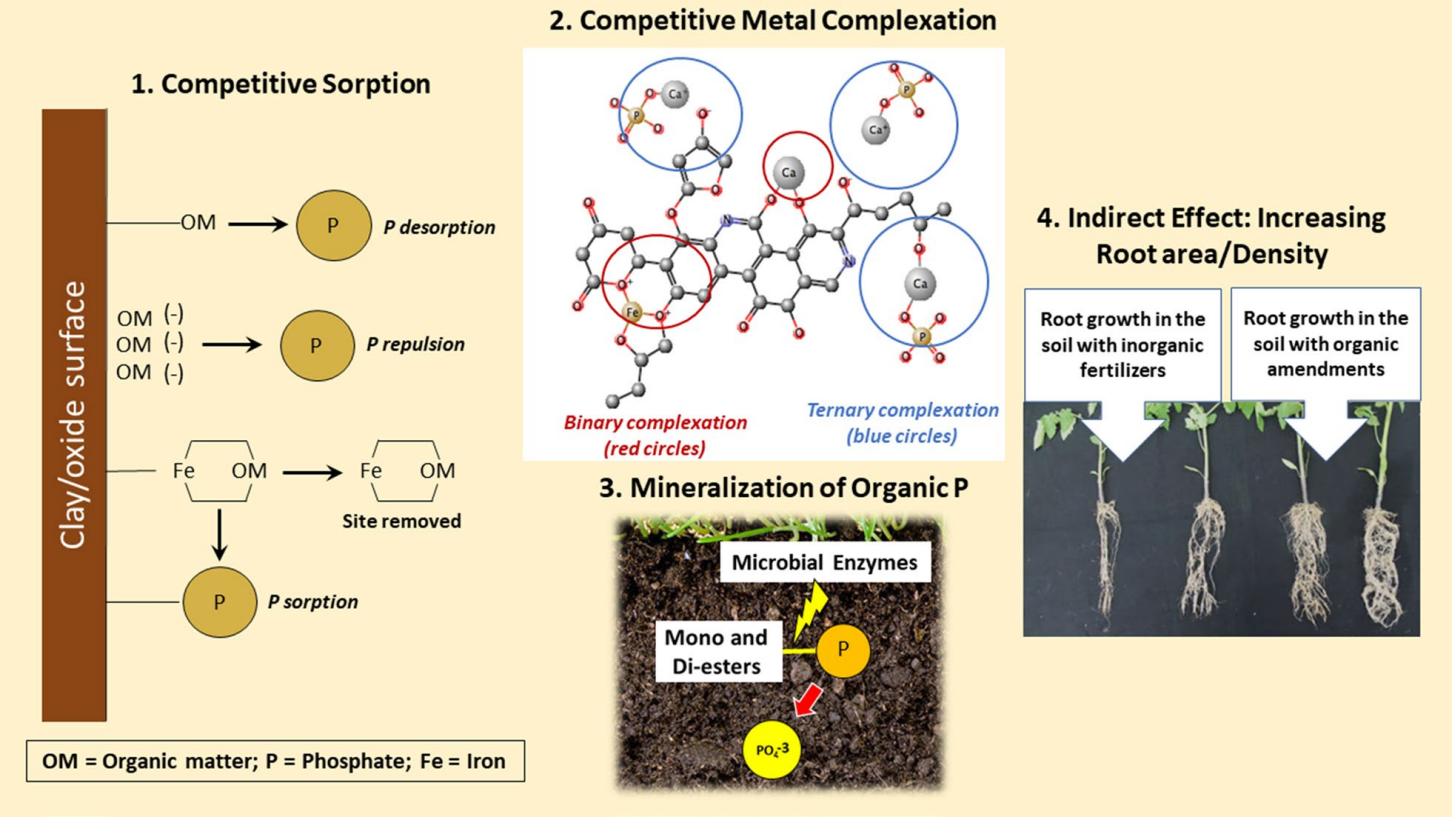

## Introduction

The soil ecosystem has a strong resilience capacity, an essential characteristic of natural ecosystems to resist changes and return to a state of equilibrium after suffering disturbance. Likewise, soil organic matter (SOM) would reach an equilibrium reflecting a certain balance between C inputs and losses if the natural (e.g., climate, topography, and soil parent materials) and human-induced factors (e.g., land-use and degradation) remain unchanged [90]. In reality, however, components of SOM continue to cycle and gradually change their properties over time as the natural and human-induced factors fluctuate, especially through the changes in climate and land management [90]. Currently, agricultural practices have been considered a source of C rather than a sink [64].

Phosphorus is an essential plant nutrient as well as N and K, thereby playing a pivotal role in the C cycle in terrestrial ecosystems [86]. It is known that plants mainly take P as a phosphate ion (i.e., H_2_PO_4_^−^ or HPO_4_^2−^ and PO_4_^3−^) from the soil, which is indicated as phytoavailable P in this review. In agricultural systems, amendments with mineral and organic fertilizers supply the P demands for crop growth and production since phytoavailable P in soil is generally very low [74]. From 2015 to 2020, the average annual growth rate of 2.3% for the global supply of P as P_2_O_5_ was applied in the soil–plant system, which is equivalent to 53 billion tons of P fertilizer [18]. However, most of the soluble P-fertilizers applied are quickly converted to forms unavailable for plant uptake. Added to the depletion of P-reserves worldwide, this places the issue of plant P-nutrition as a significant challenge to agricultural sciences and technology [73]. In addition, a large amount of P is considered to be stored with SOM; for example, storing 1000 kg of soil organic C (SOC) sequester ~ 13 kg P and ~ 22 kg P in the topsoil and the subsoil of croplands, respectively [80]. Since P is a macronutrient that limits primary production in many ecosystems, fixing a large amount of P into soils with SOM could be problematic [80]. However, the inorganic P fixed in soils as a part of SOM is unlikely as stable as P minerals fixed in soils, which could quickly become phytoavailable P [4].

**Fig. 1:**
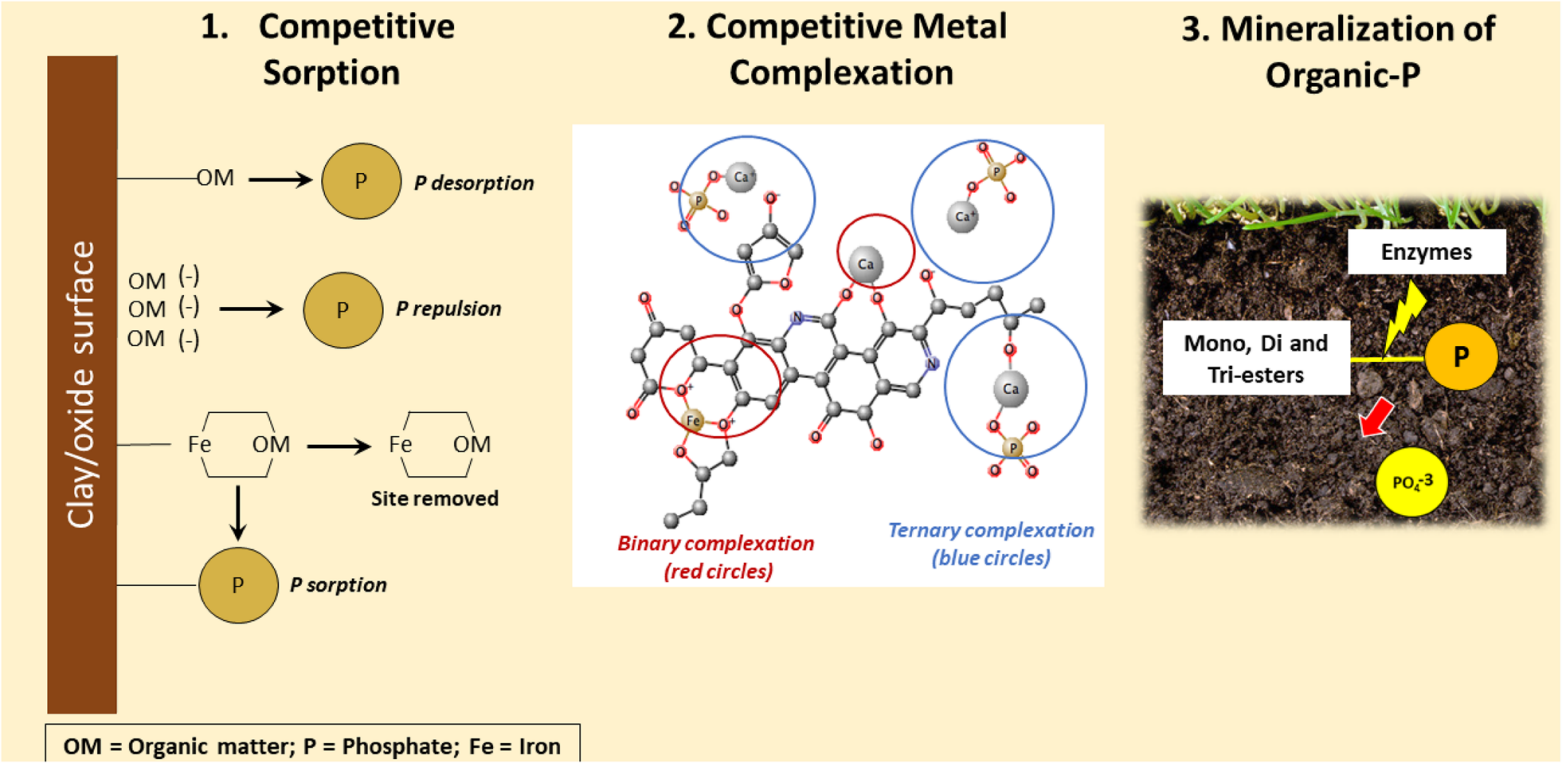
Mechanisms of the effects of organic matter on soil P chemistry. 1. Competitive sorption can occur in three scenarios: (1) OM blocking surface charge on clay or oxide mineral leads to phosphate desorption; (2) OM adsorbing on negatively charged surface of clay or oxide mineral leads to negative phosphate repulsion; (3) OM complexing with Fe adsorbed on clay or oxide mineral surface leads to the formation of Fe–OM which can be released, leaving a positively charged surface available for phosphate sorption. 2. Competitive metal complexation occurs via ternary complexation between SOM and phosphate through cations like Ca2+, depending on SOM structures. In the absence of binary complexation with either Ca2+ or Fe3+ (red circle), the negative charge on SOM is unavailable to adsorb CaPO4−. However, when the negative charge on SOM is accessible, CaPO4− can form a ternary complex (blue circle). 3. Mineralization of Organic P: microbes induce enzymatic activities to break down organic P compounds into inorganic phosphate. This process involves the degradation of mono-, di-, and phosphotriesters by specific enzymes which release inorganic phosphate for plant uptake

Several studies show that adding organic matter (OM), such as organic amendments, prevents soil P fixation and enhances phytoavailable P in soils (e.g., see Table 1 and Fig. 1). However, phytoavailable P dynamics in soils are often explained with the effects of soil pH, clay contents, and elemental compositions of calcium, iron, and aluminum in soil solution, while the contribution of SOM to phytoavailable P is often ignored. The mechanisms of SOM influencing phytoavailable P include (1) competitive sorption between SOM with phosphate for positively charged adsorption sites of clays and metal oxides (abiotic reaction), (2) competitive complexations between SOM with phosphate for cations (abiotic reaction), (3) competitive complexations between incorporation of P by binary complexations of SOM and bridging cations with the formation of stable phosphate minerals (abiotic reaction), (4) enhanced activities of exoenzymes, which affects soil P dynamics (biotic reaction), (5) mineralization/immobilization of P during the decay of SOM (biotic reaction), and (6) solubilization of inorganic P mediated by organic acids released by microbes (biotic reaction) (Fig. 1).

**Table 1:** Examples for the observations reported for the competitive sorption and complexation reactions between organic matter (OM) and phosphate ions (P)

Reactions	Forms and origin of OM	Observations	References
Competitive sorption			
Organic matter (i.e., dissolved OM and OM fractions, such as fulvic and humic acids) *inhibits P sorption* in soils	Dissolved organic matter (DOM) derived from green manure, poultry manure and cattle manure	P sorption was only inhibited by DOM derived from green manure in the order of citric acids > clover vetch • Citric acids and clover vetch can react with soil Al through ligand exchange reactions • DOM derived from animal manure has a higher molecular weight, which is not able to react with soil Al	[60]
	DOM derived from crop residues, animal manure and composts	Pre-adsorbed DOM onto mineral adsorption sites decreased P adsorption by 19%, but did not increase plant-available P Only aromatic molecules > 600 Da in DOM competed with P for mineral adsorption sites	[12]
	DOM extracted from fresh and decomposed agricultural residues	Addition of decomposed DOM decreased P sorption onto goethite, gibbsite and kaolin more than DOM derived from fresh OM The DOM adsorbed in the order of amorphous Al compounds > amorphous Fe compounds > goethite	[38]
	Fulvic acids (FA) derived from decomposing OM	Addition of FA decreased in the P sorption in three of the four soils	[35]
	Humic acids (HA) derived from soil	Addition of HA significantly decreased P adsorption onto goethite up to 28%	[20]
	Commercial HA, which contain negligible amount of total P and 36.7 g kg^−1^ carbon	Addition of HA inhibited the P adsorption in black soils	[100]
Organic matter (i.e., dissolved OM and OM fractions, such as fulvic and humic acids) *increases P sorption* in soils	DOM derived from leachate from incubated soybean and Rhodes grass hay (decomposed OM)	Addition of DOM decreased the P sorption after 1.5 h but increased P sorption in Oxisols after 6 d	[35]
	SOM in top soils was chemically removed by H_2_O_2_	The removal of SOM from topsoil resulted in a decrease in the P sorption capacity in sandy soils	[14]
Competitive complexation			
Formation of *binary complexes*: Preferrable formation of OM–Fe(III) complex over Fe–P mineral formation	Leonardite HA	Formation of Fe–P precipitation was inhibited in the presence of HA	[71]
	Aquatic OM (Suwannee River natural OM)	Stable Fe(III)-OM binary complexes prevented reaction with P and strongly suppressed the formation of Fe–P minerals (FePO_4_), the ternary OM–Fe–P complex as well as Fe(III) hydroxides	[84]
DOM inhibits CaP precipitation and transformation	HA	Formation of Ca–P precipitates was slower in the presence of HA HA strongly stabilized amorphous CaP (ACP) delaying the transformation to thermodynamically more stable phases	[22]
Formation of *ternary complexes*: Formation of OM–Ca–P complexes	Organic and mineral soil layers in four calcareous forest soil profiles	The colloidal P was originated from the overlying organic soil horizons and Ca^2+^ drove the formation of ternary SOM–Ca–P complex	[93]
	HA derived from calcareous and muck (organic) soils	Ca^2+^ preferentially complexed with HA over forming Ca–P minerals Formation of ternary HA–Ca–P complex depends on soil pH and the structures of HA	[4]
	Natural organic matter (NOM) derived from Suwannee Rover, Nordic Lake and Pony Lake	Ca–P precipitation was enhanced in the presence of NOM in the electrochemical P recovery system Transformation of ACP to stable Ca–P mineral form was delayed	[44]
	Development of organic complexed superphosphates (CSP)	Organically complexed superphosphate (CSP), i.e., monocalcium phosphate complexed by the organic chelating agent through Ca bridges, was more efficient than super phosphate in providing available P for wheat plants due to the ability of CSP to inhibit P fixation in soil	[17, 32, 33]
Formation of OM–Al(Fe)–P complexes	Humus soil samples from two groundwater discharge areas, which contain high native amounts of Al and Fe	The accumulation of Al and Fe in humic soils increased P sorption capacity by forming OM–Al(Fe)–P complexes	[29]
	HA derived from soil	Formation of HA–Al–P, but not with Fe in acidic agricultural mineral soil (pH 5.4) was observed The binding of organic P to HA does not involve either Fe or Al bridges	[8]
	HA and ferrihydrite (FH)	Complexation of FH–HA–P depended on pH and ionic strength The adsorption of P decreased with increasing ionic strength at pH < 7.5, while it increased with ionic strength at pH > 7.5 HA retarded the reduction rate of P adsorption onto the FH–HA complexes	[91]
	Metal–organic frameworks prepared with Fe and terephthalic acid (H_2_BDC)	Fe-based metal–organic frameworks (MOF) incorporated P forming OM–Fe–P complexes in eutrophic water samples Fe-based MOF exhibited a higher selectivity towards P over Cl^−^, Br^−^, NO_3_^−^ and SO_4_^2−^	[98]

We evaluate the abiotic and biotic mechanisms showing how OM or SOM influences phytoavailable P in soils.

## Abiotic mechanisms

### Competitive sorption reactions

The competitive sorption reactions between low molecular weight organic acids and phosphate were previously covered in the review by Guppy et al. [34], and the role of redox chemistry is discussed for similar systems in the context of wastewater-phosphate recovery by Wilfert et al. [97]. Here, we discuss the competitive sorption reactions for mainly other OM, such as dissolved organic matter (DOM) and SOM, including humic acids (HA) and fulvic acids (FA).

A range of organic amendments to soils, crop residues, animal manures, and other organic fertilizers have been shown to enhance phytoavailable P in soils [12, 38, 48] by reducing the phosphate adsorption while enhancing desorption of phosphate [100, 101]. Negatively charged OM is readily adsorbed onto the surfaces of positively charged clays and oxide minerals [75], thereby blocking adsorption reactions by negatively charged inorganic and organic P compounds. The sorbed OM can as well increase the repulsion of phosphate [38], thereby promoting an increase in phytoavailable P in soils (Fig. 1a). The nature of these competitive sorption reactions between SOM and phytoavailable P is summarized in Table 1.

In contrast, other studies have shown that OM additions to soils can increase phosphate sorption, decreasing phytoavailable P in soils (e.g., see Table 1). However, Guppy et al. [35] observed that the inhibition of phosphate sorption by the addition of DOM derived from decomposing OM was only short term (i.e., < 6 d). In addition, the authors suggested that previous studies of the inhibition of phosphate sorption by adding OM, such as DOM, HA, and FA, could be attributed to the phosphate contained within these OM sources [35]. However, in a study by Hunt et al. [38], which accounted for the phosphate contained in the amendment, inhibition of phosphate sorption onto metal hydroxides (i.e., goethite and gibbsite) by the addition of DOM extracted from plant biomass and dairy manure was shown.

This discrepancy in these contrasting findings can be explained mainly by the various structures of either OM molecules or the sorbing surface [38] and the availability of di- or poly-valent cations in the soils [26]. For example, the adsorption of DOM onto Fe-oxides (i.e., goethite) is mainly through multiple carboxylic functional groups (> 3) located closely spaced in the DOM and structurally specific H-bonding interactions [46]. In addition, phosphate bound to OM is mainly observed in the heavy OM fraction, which refers to organic material bound to mineral matter (i.e., up to 90% of total OM) [1], which means phosphate is likely bound binary OM–cation complexes [1], i.e., OM complexing with cations such as Fe^3+^, Al^3+^, Ca^2+^and Mg^2+^. Binary OM–cation complexes would incorporate phosphate [27], forming ternary complexes resulting in increasing phosphate sorption capacity (e.g., see Fig. 1b). We will discuss these mechanisms in the next section.

### Competitive complexation reactions

#### Binary complexations between OM and cations

Di- and poly-valent cations, such as Ca^2+^, Mg^2+^, Al^3+^, and Fe^3+^, complex with OM to form binary complexes [26]. The formation of the binary complex is strongly influenced by C functional group ionization and molecular conformation, both of which are determined by the pH of the soil solution [21]. These di- and poly-valent cations complex with mainly carboxyl and phenolic functional groups in SOM [9, 41, 69]. Phenolic functional groups, especially those containing two or more OH groups on adjacent positions of the aromatic ring, chelate multivalent metal ions, such as Fe^3+^ and Al^3+^ [81], while, Ca^2+^ is assumed to bind mainly to carboxyl functional groups [54]. Previous studies reported that an electrostatic attraction plays an essential role in the bonding between carboxyl functional groups and either ferrihydrite (FH) [61] or Ca^2+^ [4].

High molecular weight OM consisting of hydrophobicity and aromatic structures can remarkably stabilize SOM [63, 79], thereby reducing mineral formations by enhancing stereochemical effects on blocking active crystal growth sites [37]. The formation of binary complexes is known to delay the transformation of labile inorganic phosphate forms, not strongly adsorbed in the soil. As a result, it may become phytoavailable P relatively quickly to stable phosphate minerals, including Ca–P or Fe–P minerals, such as apatite and strengite, respectively. Examples of these observations are summarized in Table 1. The following mechanisms could explain these reactions. Firstly, kinetically favored labile Ca–P minerals, such as brushite, can overgrow onto adsorbed surfaces of SOM fractions, including HA and FA [31], and thereby transformation of labile Ca–P to stable Ca–P is delayed. Secondly, SOM consisting of multiple negative-charge domains of phenolic or carboxyl functional groups either re-structure stable phosphate minerals such as apatite or strengite into a sponge-like structure or re-crystallize into more labile hydrated structures [101]. Lastly, the complexation of OM and FH is enhanced in the presence of Ca^2+^, especially pH above 7, by forming FH–Ca–OM ternary complexes, thereby decreasing the formation of stable Fe–P and Ca–P minerals [78].

#### Ternary complexations of OM, cations, and P

Organic matter forms binary complexes with cations and ternary complexes with anions, including phosphate, via cation bridging [24, 78]. Levesque and Schnitzer were the first to report the ternary formation of FA–metal–P complexes in 1967 [72]. Since then, potential formations of ternary complexes among OM fractions, P, and cations such as Fe^3+^, Al^3+^, and Ca^2+^ have been observed in agricultural lands, calcareous soils, wastewater treatments, and eutrophic lakes (e.g., see Table 1).

Wang et al. [91] observed that the amounts of phosphate incorporated by the binary HA–FH complexes significantly decreased (i.e., up to 60%) as pH increased from 3.5 to 9.5. The reduction in the incorporated phosphate with a rising pH is mainly due to the alteration in the surface charge of the binary HA–FH complexes, i.e., the binary complexes becoming more negatively charged at alkaline pH [91]. Additionally, there is a change in the phosphate species, i.e., phosphoric acids mainly exist as H_2_PO_4_^−^ in soils at pH below 7.2 and HPO_4_^2−^ at pH above 7.2 (*pK*_a2_ = 7.2 when *I* = 0.0 M and at temperature 25 °C), thereby causing more electrostatic repulsion towards the negatively charged HPO_4_^2−^ as pH increases. Audette et al. [4] observed that HA extracted from two types of soils (i.e., riparian soil, RSHA, and organic agricultural soil, OAHA) complexed with Ca^2+^ more at pH 8.5 than 6.0. However, there was no significant difference in the amount of phosphate incorporated by the binary OAHA–Ca complex between pH 6.0 and 8.5.

On the other hand, the amount of phosphate incorporated by the binary RSHA–Ca complex at pH 6.0 was higher than that at pH 8.5 [4]. The authors concluded that the ternary complexation of HA–Ca–P form when forming binary HA–Ca complex is through either monodentate complexation or electrostatic attraction. In contrast, the ternary complexation does not form when the binary HA–Ca complex forms through bidentate complexation. Thus, the formation of the ternary OM–cations–phosphate complex is highly dependent on the structures of OM, i.e., the location of acidic functional groups and pH [4, 6, 91]. Formation of ternary complexation between phosphate and Fe–OM is considered to be six to seven times larger than amorphous Fe-oxide, where the mole fraction between the incorporated phosphate and Fe in Fe–OM binary complex is close to one [27].

Organic superphosphate fertilizer, which is the complex of monocalcium phosphate and HA, increases an agronomical P-use efficiency by lowering soil P fixation (see the studies by [17, 36, 89]). In addition, this organic fertilizer is water-soluble because the complex only involves one cation (i.e., monocalcium phosphate, CaPO_4_^−^) [89] and can lower soil P fixation since phosphate in the ternary complex can be quickly mobilized by root exudate organic anions or upon acidification [25].

Although the ternary complexed-phosphate could account for more than 50% of the dissolved phosphate in natural water and soil solution, the ternary complexed-phosphate is often ignored because this complexed-phosphate is not detected by the conventional phosphate speciation procedures where up to 80% of OM-associated phosphate is hydrolyzed and become free P [24].

As we discussed the abiotic mechanisms, it is clear that SOM could control P mobilization and enhance phytoavailable P in soils by delaying soil phosphate fixation, even though the formation of binary or ternary complexations depends on the structures of OM and soil pH. Therefore, these mechanisms should be explored further to enhance phytoavailable soil P and soil health by increasing SOM.

## Biotic mechanisms

### Enzymes in the P cycle

Inositol phosphates, like mono-, di-, and phosphotriesters, are the primary sources of P for biomolecules. These forms of inositol phosphate are commonly found in complex organic matter in soils [88]. A portion of the P released from organic compounds is integrated into the microbial biomass, an essential P reserve protected from soil mineral fixation [82].

Hydrolytic enzymes, which are involved in nutrient cycling, can serve as indicators of soil biochemical status. Enzymes are closely associated with the cycling and accessibility of inositol phosphate and its various forms in soils. Microbes, including bacteria and fungi, play a vital role in transforming and cycling inositol phosphate and its derivatives in soils. These microbes secrete enzymes, such as phosphatases, which hydrolyze inositol phosphate esters to release free phosphate, a form that plants can quickly absorb. This mineralization process releases phosphorus and produces other organic molecules, including inositol and glucose, that can serve as energy sources for soil microbes. Overall, enzymes from soil microbes are critical for cycling and availability of inositol phosphate, impacting plant growth and ecosystem productivity [23].

Given that each enzymatic activity relates to a specific substrate and a particular reaction [53, 57], the measurements of several enzyme activities must be included for a more profound knowledge to address the general state of nutrients in the soil. Furthermore, most organic substrates entering the soil are complex polymers and need to be decomposed by extracellular enzymes so that the simple monomeric compounds can be taken up and used to support the metabolic processes of soil microorganisms [58]. Therefore, the studies of different hydrolase enzyme activities are essential since they indicate the potential of soil to carry out specific biochemical reactions and maintain soil fertility.

Four representative soil enzymes are related to the soil P cycle; *Acidmonophosphoatase*, *Alkalinemonophosphatase*, *Phosphodiesterase*, and *Phytase*, which are frequently studied [15]. They are a vital catalyzer in mineralizing organic P compounds into inorganic P as a form of phytoavailable P, which plants assimilate. These extracellular enzymes are secreted by bacteria, fungi, protozoa, or root exudates [56]. While fungi mainly produce acid phosphatase, alkaline phosphatase is generated by bacteria [15]. The activities of these enzymes and their inhibition are strongly influenced by the concentration of orthophosphates in soil.

Soil physicochemical properties are also key factors to optimize environmental conditions for those enzymes, such as pH, soil texture, and content of OM. With the P demand from microbes and plants, climate regulates the intensity of these enzyme activities. For example, Margalef et al. [49] reported that the global trend of acid phosphatase, which is strongly related to mineralization, varies largely by annual precipitation and temperature. At higher temperatures, phosphatase activities and higher microbial activities are high compared to low temperatures. Additionally, these authors stressed the importance of total soil N content as a fundamental resource of the enzymes [49].

Some plant species also produce phosphatase through roots, particularly under conditions of soil P deficiency. For example, white Lupin (*Lupinus albus* L. cv*. Kievskij mutant*) is known to induce acid phosphatase, alkaline phosphatase, phytase, and chitinase from cluster root (CR) exudates by releasing carboxylic acids for mobilizing organic P [51, 94]. Also, other enzymatic activities and their gene expression levels, including citrate synthase, malate dehydrogenase, and phosphoenolpyruvate, have been reported to increase in lupin CR [68]. Soil P deficiency often coexists with high Al content in acid soils, and lupin CR exudates display different strategies: 1 limiting the entrance of Al into the roots through the formation of non-toxic complexes, 2 mobilizing the toxic metals; and 3 secreting Malate and citrate for Al resistance [68].

Moreover, *Burkholderia* species, an N fixation bacteria, are dominant inhabitants in the white lupin root [96] and are regarded as a source of acid phosphatase activity [95].

### Interaction between SOC and enzymes

The primary soil enzyme sources are roots, animals, and microorganisms. Once enzymes are produced and excreted from microbial or root cells, they face harsh conditions. Most are rapidly decomposed by organisms [10], and the remainder is adsorbed to soil organo-mineral colloids and possibly protected against microbial degradation [2]. The fraction of the soil extracellular enzyme activity, which is not denatured or inactivated through interactions, is referred to as immobilized [28]. Immobilized enzymes in soil are protected by two associations: (1) interaction with inorganic mineral colloids like clay surfaces and (2) association with organic complexes such as humic molecules. These interactions enhance the resistance of extracellular enzymes to thermal and proteolytic degradation [56]. Adding organic amendments can further strengthen this protection [39].

However, it has been reported that immobilization with humic substances can reduce enzymatic activity or even act as an inhibitor [2, 43, 52]. For example, humic substance molecules have been found to block some enzyme-active sites (Mazzei et al. 2013). The inhibitory effects may be counterbalanced by enhanced stability and resilience of enzymes towards decomposition, elevated temperature, and/or proteolysis [45]. The underlying mechanisms through which immobilization processes stabilize enzymes and retain their activity are not fully understood. The possible significant mechanisms include electrostatic interaction and hydrophobicity [45], which are influenced by factors such as pH, ionic strength, and HS-protein complexation.

To better understand the underlying causes of electrostatic interaction and hydrophobicity, it is necessary to consider a wide range of factors. These include the particle size of humic substances (HA vs. FA) [45], pH range [62], the molecular weight of each enzyme [5], kinetic behaviors (e.g., Michaelis–Menten constant and maximal velocity) [13], and mass ratio between humic substance and enzyme protein [45].

Immobilized enzymes and complex humic synthesis can be practical tools for plant nutrient uptake by encapsulating seed coatings [55, 87]. By engineering the encapsulation of seed coating with the enzyme-humic complex, the immobilized phosphatase, which is resistant to degradation, can directly provide phosphorus uptake to plants [65–67] leading to higher P content in shoot tissue.

### Solubilization of inorganic P mediated by microbes

Microbial communities can play pivotal roles in P dynamics in the biosphere. Increases in SOM, which have more available nutrients for microbes, enhance the microbial biomass and microbial activity of soils and result in higher P turnover by microbial immobilization of inorganic P, mineralization of organic P, and microbial P synthesis [16, 42]. Another function of SOM is protecting bounded enzymes in the humic matrix against degradation, such as high temperature [66]. Several studies have shown that the relative abundance of soil microbes and their activities was increased by applying organic amendments (e.g., [40, 47, 92]).

P immobilization and mineralization depend on the soil's inherent C-to-P stoichiometry [83]. The ratio of C to P can be increased by adding organic amendments such as crop straw and cover plants [102]. Xu et al. [99] showed that the concentration of excess phytoavailable P in the topsoil was reduced due to an increase in microbial immobilization of phytoavailable P into microbial biomass driven by the addition of SOC. In natural terrestrial systems, the mineralization of organic P is the primary microbial process that increases soil phytoavailable P [74]. Organic mineralization is a P-source accessed by several bacteria and fungi [82]. Plant residues, animal manures, and humus are sources of organic P mineralized to phytoavailable P by microbial phosphatases and phytases exoenzymes.

The following mechanisms represent the microorganism’s involvement in soils with the solubilization of P.

#### Acidification

Soil-borne microbial communities can release phytoavailable P by inorganic phosphate solubilization from primary minerals (i.e., apatite), secondary compounds formed by the precipitation of soluble P-forms (i.e., Ca–P, Fe–P, Mn–P and Al–P minerals) and P-desorption from the surfaces of clays, Fe- or Al-oxides and carbonates. In addition, soil P-mining strategies can be amplified using P-solubilizing microorganisms (PSM) that include representatives from several species of bacteria and fungi [3].

The primary mechanism responsible for the release of P from mineral sources is the acidification coupled with a chelation process mediated by gluconic acid, oxalic acid, citric acid, fumaric acid, malic acid, lactic acid, isovaleric acid, acetic acid, 2-ketoglucoronic, among other secreted organic acids [3, 50]. The amount of phosphate solubilization depends on the microbial species, the availability of the C source, the secreted organic acid, and the cation bound to P [50].

#### Chelation

In addition to acidification, chelation is another mechanism that microorganism display by producing a chelating agent formed with small organic molecules, so-called “siderophores” [59]. This chelating agent binds to phosphate ions and creates a more stable complex to avoid the formation of insoluble P in soil. Some bacterial species, for example, can produce siderophores, such as rhizobactin and pseudobactin, that have been shown to enhance the solubilization of phosphorus in soil [76].

#### Enzymatic hydrolysis

Enzymatic hydrolysis is another mechanism by which P-solubilizing microorganisms can solubilize P in soil, and some fungi can produce enzymes that release phytoavailable P from organic matter. Arbuscular mycorrhizal fungi (AMF) are a well-known group that produces acid and alkaline phosphatase. It should be highlighted that hydrolytic enzyme production can vary depending on the fungal species and the environmental conditions in which they grow. Some fungi may be more efficient at solubilizing P than others, and the activity of these enzymes can be affected by factors such as soil pH, temperature, moisture, and nutrient availability [39]

## Fertilisation and management strategies

Currently, several strategies based on microbial technologies have been designed to increase the agronomic effectiveness of P-fertilizers in terms of P-use efficiency for crop production (e.g., [3, 19, 70, 77, 85]). A combination of technologies can be used to increase phosphate solubilization activity. Among them is the design of a consortium with elite strains of bacteria and fungi and combining proper PSM bioinoculant formulation and delivery. The most relevant approach is for PSM to be combined with rock phosphates and stable OM or its fractions (i.e., HA and FA) to reduce P-adsorption and precipitation on the mineral lattice [3, 7, 30, 70, 77].

Microbial formulations combining P-solubilizing and P-mineralizing properties have been proposed in association with OM and slow-release mineral P-sources [7, 11, 30, 77] as a suitable P-fertilizer complementary to fully acidulated inorganic P-sources. Reductions in phytoavailable P source application rates (e.g., single superphosphate, SSP) can be achieved without compromising crop productivity. For example, Giro et al. [30] applied 60% of rock phosphate and 40% of SSP and, combined with MSP and HA, showed increased maize biomass by ~ 20% compared to plants that received the fertilizer.

Biofortification of plant substrates and the design of organo-mineral fertilizers with different P-release rates can be obtained by biological enrichment of composts and vermicomposts of various raw materials, and in the presence of rock-phosphate generates a value-product with increased contents of phytoavailable P [11]. Therefore, a new generation of P-biofertilizers should consider a selection of a microbial consortium with a P-release complementary mechanism, a slow P-release source, combined with readily soluble and stable OM to increase the P-use efficiency for different cropping systems.

## Conclusion

We reviewed the biotic and abiotic mechanisms of the sorption/desorption, precipitation/dissolution, and mineralization/immobilization of P affected by SOM and P's fertilization and management strategies based on microbial technologies. The phytoavailable P in soils can be increased by the results of (1) competitive sorption onto the negatively charged surfaces of either clay or (hydro)oxides, (2) competitive complexation with di- or poly-valent cations such as Ca^2+^, Mg^2+^, Al^3+^, or Fe^3+^, (3) enhanced mineralization of organic P and solubilization of inorganic phosphate minerals by enzymes or microbes. Soil organic matter can not only increase phytoavailable P in soils but also preserve enzymes through their physical interaction and protection against degradation in the long run. However, some cases report the opposite effects (e.g., reduction and inhibition of enzymatic activities). Conversely, the phytoavailable P in soils may be decreased in the short-run by the results of (1) increasing positively charged sites provided by SOM, (2) adsorption onto SOM via cation bridging, (3) complexation of ternary complexes (i.e., SOM–cation–phosphate), and (4) enhancing immobilization of P by microbes as well as SOM and clay fractions.

These mechanisms highly depend on the structures of SOM, soil characteristics such as pH, the composition of mineral matter, the ratios of C/N/P, and the proliferation of microbes. A better understanding of the mechanisms of how SOM influences the phytoavailable P in soils is required for establishing effective agricultural management for soil health and enhancement of soil fertility, especially for increasing P-use efficiency. Soil organic matter has a critical role in regulating soil P dynamics and the production of phytoavailable P in soils, and therefore the biotic and abiotic contributions of SOM on phytoavailable P in soils should be further explored.

## Data Availability

Not applicable.

## Abbreviations

Term: Meaning
SOM: Soil organic matter
SOC: Soil organic carbon
OM: Organic matter
DOM: Dissolved organic matter
DOC: Dissolved organic carbon
HA: Humic acids
FA: Fulvic acids
FH: Ferrihydrite
PSM: P solubilizing microorganisms
SSP: Single superphosphate
